# Prognostic relevance of global work index and global constructive work in patients with non-ischemic dilated cardiomyopathy

**DOI:** 10.1007/s10554-024-03144-5

**Published:** 2024-05-23

**Authors:** Peng Chen, Matthias Aurich, Sebastian Greiner, Gabriele Maliandi, Matthias Müller-Hennessen, Evangelos Giannitsis, Benjamin Meder, Norbert Frey, Sven Pleger, Derliz Mereles

**Affiliations:** Department of Cardiology, Angiology and Pneumology, University of Heidelberg, Im Neuenheimer Feld 410, 69120 Heidelberg, Germany

**Keywords:** Dilated cardiomyopathy, Echocardiography, Global longitudinal strain, Myocardial work, Survival

## Abstract

Myocardial work (MW) derived from pressure-strain loops is a novel non-invasive tool to assess left ventricular (LV) function, incorporating global longitudinal strain (GLS) by speckle tracking echocardiography and non-invasively assessed blood pressure. Studies on the role of MW in dilated cardiomyopathy (DCM) are still limited. Therefore, the aim of this study was to evaluate the potential value of MW for predicting adverse outcomes in patients with DCM. 116 consecutive patients with DCM who underwent heart catheterization were retrospectively recruited from June 2009 to July 2014. 34 patients (30%) met the composite endpoints for major adverse cardiac events (MACE) of cardiac transplantation, need for implantable cardioverter-defibrillator (ICD) therapy, heart failure hospitalization and all-cause mortality. Patients with DCM were followed up for a mean of 5.1 years (IQR: 2.2–9.1 years). Global work index (GWI) and global constructive work (GCW) were not only independent predictors but also provided incremental predictive values (Integrated discrimination improvement [IDI] > 0) of MACE in multivariate Cox models. Furthermore, Patients with GWI < 788 mm Hg% (HR 5.46, 95%CI 1.66–17.92, *p* = 0.005) and GCW < 1,238 mm Hg% (HR 4.46, 95%CI 1.53–12.98, *p* = 0.006) had higher risks of MACE. GWI and GCW assessed by strain imaging echocardiography may have an additional value beyond LV-EF and GLS for predicting adverse outcomes in DCM.

**Supplementary Information:**

The online version contains supplementary material available at 10.1007/s10554-024-03144-5.

## Introduction

Dilated cardiomyopathy (DCM) is a progressive disease characterized by left ventricular (LV) dilation and contractile dysfunction that eventually develop to heart failure (HF) or death [1]. Previous studies have shown that the 5-year mortality rate for appropriately treated patients with DCM in systolic heart failure ranges from 21–28% [2, 3]. Therefore, an in-depth understanding as well as proper assessment of LV function is particularly important for diagnosis and prognosis assessment of DCM.

LV ejection fraction (LV-EF) is widely used as the first-line tool to assess the LV performance. However, it has limitations: (1) it has a wide inter-observer variability leading to lower accuracy and reproducibility; (2) it is load- and geometry-dependent, thus reflecting LV function only partially, e.g. in dilated or hypertrophic cardiomyopathy, and (3) it does not represent a measure of myocardial contractility [4, 5].

Over the past decades, global longitudinal strain (GLS) by speckle-tracking (STE) echocardiography, which have a good inter- and intraobserver reproducibility, became a new method to assess LV function [6]. Moreover, it has become more widely used in clinical practice because of its high sensitivity in detecting subtle changes of LV contractile performance. However, it has been shown that elevated afterload can cause a decrease in GLS, which can lead to an incorrect assessment of myocardial deformation, implying a load dependency similar to LV-EF [7].

In recent years, myocardial work indices (MWI) derived from left ventricular pressure-strain loop (LV-PSL), integrating GLS and peak LV systolic pressure by cuff manometer, has been used as a novel method to assess LV performance. The MWI include global work index (GWI), global constructive work (GCW), global wasted work (GWW) and global work efficiency (GWE) [8]. Russell et al. first demonstrated that PSL using non-invasive pressures correlated well with invasive assessment in patients with left bundle branch block and dyssynchrony [9]. In addition, MWI may be more sensitive in detecting altered loading conditions. For instance, GWI in patients with systemic arterial hypertension was significantly increased, while GLS and LV-EF had no significant changes compared to healthy controls [10].

MWI consider deformation and afterload, and its diagnostic and prognostic value has been validated in several hemodynamic conditions [10–13]. However, there are limited studies on MWI in patients with DCM, especially in terms of prognosis. Therefore, the purpose of this study was to evaluate potential value of MW indices for predicting adverse outcomes in patients with DCM.

## Methods

### Study population

169 consecutive patients with impaired LV function who underwent heart catheterization from June 2009 to July 2014 were retrospectively included. Patients were defined as having a LV-EF < 45% assessed by echocardiography, in the absence of coronary and/or moderate to severe primary valvular heart disease. Patients with non-dilated DCM were also included [14]. 53 patients were excluded due to poor image quality hindering deformation analyses. Finally, 116 DCM patients were analyzed. This study was conducted after approval by the Ethics Committee of the University of Heidelberg, in accordance with the current version of the Declaration of Helsinki and with informed consent of all participants.

### Echocardiography

All echocardiographic examinations were performed on a commercially available ultrasound machine (Vivid E9, GE Healthcare Vingmed, Trondheim, Norway) according to the guidelines of the American Society of Echocardiography, using a 1.5–4.6 MHz phased array probe (M5S-D) [15]. Two-dimensional, color Doppler, pulsed-wave and continuous-wave Doppler data, as well as GLS and MWIs were analyzed offline using the EchoPAC software (EchoPAC workstation BT13, GE Healthcare, Trondheim, Norway).

### Global longitudinal strain

Images were acquired from the apical 4-, 2- and 3-chamber views with a frame rate of at least 50 fps. GLS by STE was assessed offline. The software tracks the endocardial borders automatically, building the region of interest (ROI), which can be adjusted manually if necessary. Weighted average values from the 17 segments are calculated and a polar map (bull’s eye) is built.

### Myocardial work

MW was assessed by incorporating systolic and diastolic blood pressures measured by a cuff. The peak systolic pressure is a fair approximation of central aortic pressures and of systolic LV pressures in the absence of a gradient through the left ventricular outflow tract and the aortic valve. Subsequently, according to the duration of ejection and isovolumetric phases defined by the timing of mitral and aortic valve opening and closure, the software generates a non-invasive pressure strain loop [16]. Following MW were calculated:

1. GWI: represents the average of total work in systole, approximately equals to the area of PSL from mitral valve closure to mitral valve opening.
2. GCW: it represents the work performed by LV myocardial shortening during systole and lengthening during isovolumetric relaxation.
3. Global wasted work (GWW): it represents the work performed by LV myocardial lengthening during systole and shortening during isovolumetric relaxation.
4. Global work efficiency (GWE): it represents the percentage of GCW divided by the sum of GCW and GWW.

### Clinical outcomes

Follow-up was obtained by review of the digital clinical records, using the time of the patient’s last follow-up visit in the hospital assessed until December 2022. In this study, major adverse cardiovascular events (MACE) were defined as: all-cause mortality, cardiac transplantation, appropriate implantable cardioverter-defibrillator (ICD) therapy, either a shock or antitachycardia pacing, and heart failure hospitalization.

### Statistical analysis

All data was analyzed using SPSS Statistics for Windows, version 26.0 (SPSS Inc., Chicago, USA). Continuous variables were expressed as mean ± standard deviation and analyzed using the Student’s t-test, or as medians (25, 75 quartiles) and analyzed by the Mann-Whitney U-test if data was non-normally distributed. P values < 0.05 were considered as statistically significant. Categorical variables were expressed as frequencies and percentages and analyzed using the Chi-square test or the Fisher’s exact test as required. Receiver operating characteristics curve (ROC) analysis was used to assess which LV functional parameter was most strongly associated with MACE with the corresponding cut-off value, sensitivity and specificity. The cut-off values for dichotomous analyses of LV functional parameter were derived by the maximum of Youden index. Subsequently, Kaplan-Meier survival analyses were conducted to compare the prognosis. Univariate Cox regression analysis was used to analyze the association between parameters and MACE in patients with DCM. Multivariate Cox regression analysis was carried out for variables with *p* < 0.05 in univariate analysis to analyze which parameter was an independent predictive factor for MACE. We used the Schoenfeld residuals to verify the proportional hazards assumption, and used the variance inflation factor to check the multicollinearity in Cox regression. In addition, integrated discrimination improvement (IDI) and the Harrell C concordance statistics (C-index) were used to assess the incremental value of predicting adverse outcomes.

## Results

### Characteristics of DCM patients

**Fig. 1:**
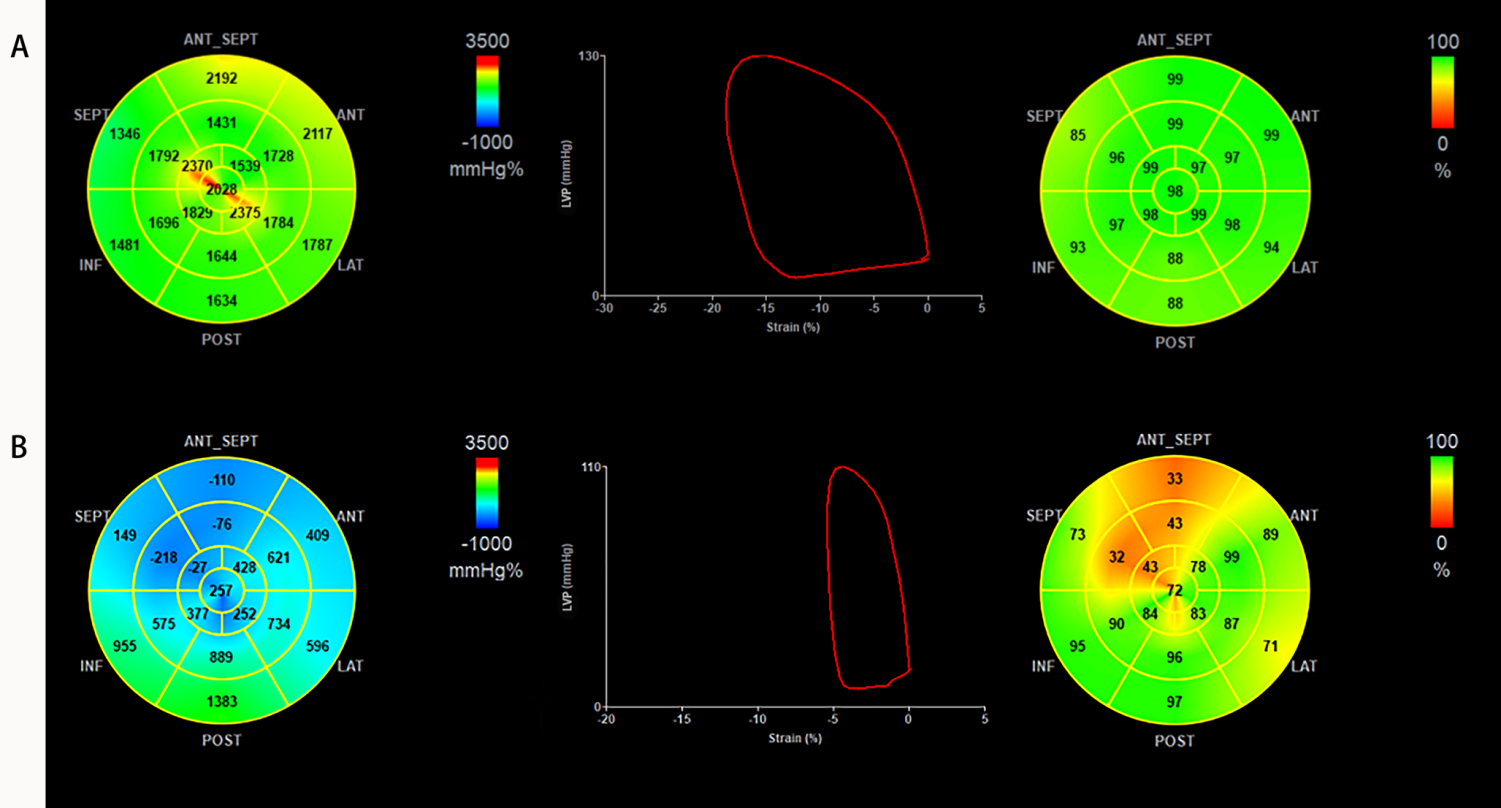
Healthy controls (panel A) and DCM patients (panel B). The left panel shows a 17-segment bull’s-eye representation of myocardial work: areas of negative work are in blue, normal work in green and high MW work in red. The middle panel shows the LV-PLS. The right panel shows a 17-segment bull’s-eye representation of GWE showing areas of high efficiency coded in green and those with the least efficiency coded in red. DCM: non-ischemic dilated cardiomyopathy, MW: myocardial work, LV-PSL: left ventricular pressure-strain loop, GWE: global work efficiency

Clinical, laboratory and echocardiography parameters of 116 patients with DCM are shown in Table 1. Graphical examples for GWI, LV-PSL and GWE in one DCM patient and one healthy control individual are shown in Fig. 1. GWI and GCW were significantly lower in patients with MACE compared with those without MACE.

**Table 1:** Characteristics of DCM patients stratified according to MACE

Parameters	No MACE	MACE	*p*
N	82	34	
**Baseline characteristics**			
Age, years	55.1 ± 14.3	55.0 ± 13.4	0.97
Male, n (%)	67 (82)	20 (59)	0.010
BMI, kg/m^2^	26.1 ± 3.7	25.3 ± 4.0	0.35
SBP, mm Hg	121.4 ± 16.5	114.4 ± 18.4	0.044
DBP, mm Hg	75.8 ± 11.2	72.1 ± 10.4	0.10
NYHA class ≥ II, n (%)	40 (49)	26 (77)	0.006
**Clinical chemistry**			
NT-proBNP, pg/mL	376 (114, 913)	1206 (502, 2516)	< 0.001
hs-TNT, ng/L	10 (6, 19)	12 (7, 46)	0.159
**Echocardiography**			
LV-EDD, mm	56.0 ± 7.2	59.8 ± 8.6	0.016
LV-ESD, mm	45.8 ± 9.2	52.7 ± 9.8	< 0.001
LV-EDV, mL	152.0 ± 49.8	184.2 ± 58.5	0.003
LV-ESV, mL	99.6 ± 41.3	137.5 ± 54.8	0.001
LV-EF, %	35.5 ± 9.8	26.9 ± 8.8	< 0.001
IVS, mm	10.9 ± 2.2	10.1 ± 2.2	0.07
PW, mm	10.4 ± 1.8	10.1 ± 2.4	0.44
LV mass index, g/m^2^	120.6 ± 31.7	132.9 ± 41.4	0.09
MAPSE, cm	1.3 ± 0.3	1.1 ± 0.3	0.006
LV-GLS, -%	12.5 ± 4.2	8.9 ± 3.6	< 0.001
GWI, mm Hg%	1,144.8 ± 403.1	759.9 ± 417.6	< 0.001
GCW, mm Hg%	1,448.3 ± 464.5	1,004.9 ± 453.4	< 0.001
GWW, mm Hg%	150 (109, 214)	154 (91, 271)	0.89
GWE, %	89 (84, 92)	84 (73, 90)	0.002
E/e’	7 (4, 10)	10 (7, 14)	0.001
LAVI, mL/m^2^	25 (21, 37)	32 (28, 42)	0.002
RV-EDD, mm	36.0 ± 6.7	36.4 ± 9.7	0.80
RV-EDA, cm^2^	17.9 ± 4.4	19.1 ± 6.9	0.35
RV-ESA, cm^2^	11.7 ± 3.4	13.3 ± 5.6	0.12
RV-FAC, %	34.2 ± 13.3	31.0 ± 12.8	0.23
RV-GLS, -%	13.8 ± 5.3	10.6 ± 4.8	0.003
TAPSE, cm	1.9 ± 0.5	1.7 ± 0.5	0.025
RA-ESA, cm^2^	15.8 ± 5.5	15.6 ± 5.0	0.83
RA-AI, cm^2^/m^2^	7.9 ± 2.8	8.3 ± 2.7	0.51

MACE, major adverse cardiac events; BMI, body mass index; SBP, systolic blood pressure; DBP, diastolic blood pressure; NYHA, New York Heart Association; NT-proBNP, N-terminal pro brain natriuretic peptide; hs-TNT, high-sensitive cardiac troponin T; LV, left ventricle; EDD, end-diastolic diameter; ESD, end-systolic diameter; EDV, end-diastolic volume; ESV, end-systolic volume; EF, ejection fraction, biplane modified Simpson’s rule; IVS, interventricular septum; PW, posterior wall; MAPSE, mitral annular plane systolic excursion; GLS, global longitudinal strain; GWI, global work index; GCW, global constructive work; GWW, global wasted work; GWE, global work efficiency; E/e’, ratio of passive mitral inflow velocity (E) to tissue Doppler mitral annular velocity (e’); LAVI, left atrial volume index; RV, right ventricle; RV-EDD, RV end-diastolic diameter; RV-EDA, RV end-diastolic area; RV-ESA, RV end-systolic area; RV-FAC, RV fractional area change; RV-GLS, RV global longitudinal strain; TAPSE, tricuspid annular systolic plane excursion; RA, right atrium; RA-ESA, RA end-systolic area; RAAI, RA end-systolic area index

### Follow-up

Patients with DCM were followed up for a mean of 5.1 years (interquartile range [IQR]: 2.2–9.1 years). 34 patients (30%) met the composite endpoints: 5 patients received cardiac transplantation, 17 patients were hospitalized due to heart failure, 9 patients received appropriate ICD therapy and 3 patients died.

None, mild, moderate and severe mitral regurgitation were present in 40 (34.5%), 60 (51.7%), 12 (10.3%) and 4 (3.5%) patients respectively. None, mild and moderate aortic regurgitation were present in 81 (69.8%), 31 (26.7%) and 4 (3.5%) patients respectively. None, mild and moderate tricuspid regurgitation were present in 58 (50.0%), 47 (40.5%) and 11 (9.5%) in patients respectively. All patients were treated according to heart failure guidelines prior to 2015, thus, without sacubitril/valsartan and without sodium-glucose transporter 2 inhibitors.

### Clinical, laboratory and echocardiographic variables associated with MACE

**Fig. 2:**
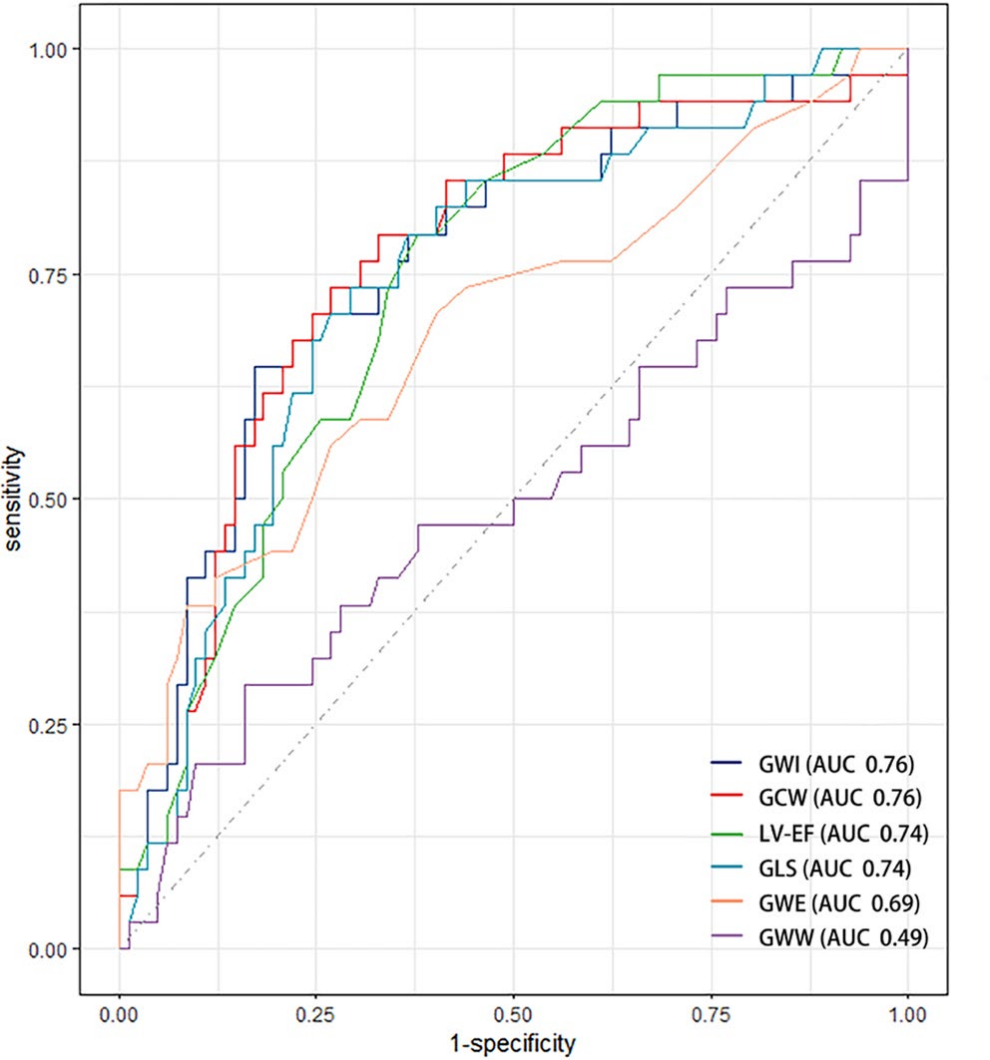
Receiver operating characteristics curve (ROC) of associations between LV functional parameters and MACE. LV-EF: left ventricular, biplane ejection fraction, GLS: global longitudinal strain, GWI: global work index, GCW: global constructive work, GWW: global wasted work, GWE: global work efficiency, MACE: major adverse cardiac events

**Fig. 3:**
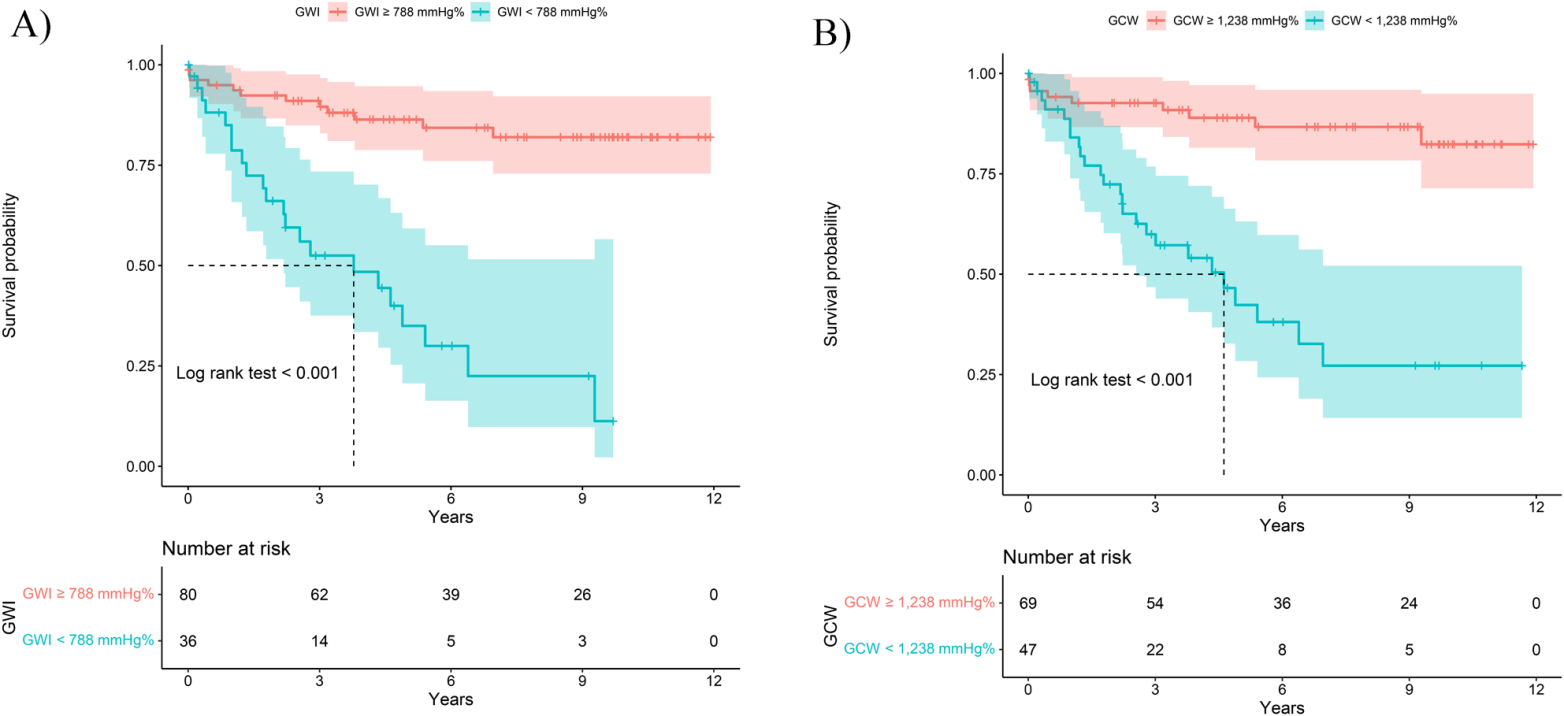
Kaplan-Meier (KM) survival curves displaying the occurrence of MACE over time. GWI: global work index, GCW: global constructive work, MACE: major adverse cardiac events

The performance for prognostication of MACE was significant for GWI, GCW, LV-EF and LV-GLS with area under curves (AUC) ranging from 0.74 (LV-EF and LV-GLS) to 0.76 (GCW and GWI) (Fig. 2). The cut-off value of GCW and GWI were 1,238 and 788 mm Hg%, with corresponding sensitivity and specificity of 73.5% and 73.2%, 64.7% and 82.9%, respectively. In the Kaplan-Meier survival analysis, patients with GWI < 788 mm Hg% (hazard ratio [HR] 6.91, 95% confidence interval [CI] 3.36–14.19, *p* < 0.001) and GCW < 1,238 mm Hg% (HR 6.40, 95%CI 2.95–13.89, *p* < 0.001) had higher risks of MACE (Fig. 3).

**Fig. 4:**
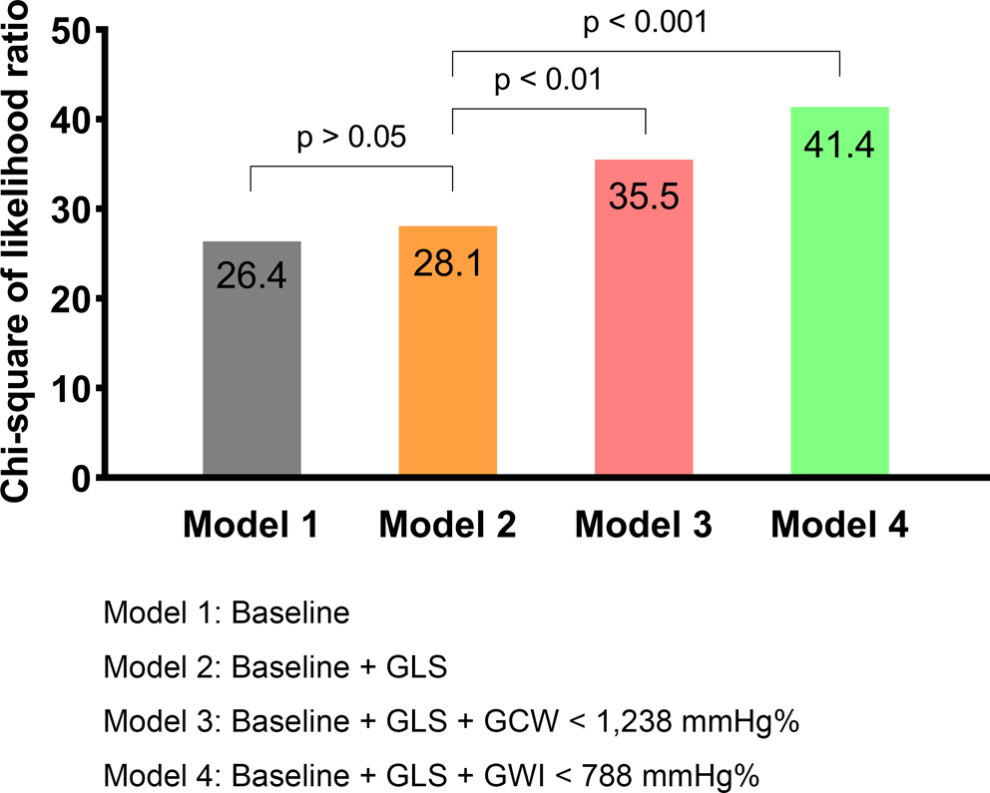
Comparison of multivariate Cox regression models. Baseline includes NYHA class (≥ II), NT-proBNP > 681, E/e’, LV-EF. GLS: global longitudinal strain, GWI: global work index, GCW: global constructive work

The results of univariate Cox regression analysis are shown in Table 2. Subsequently, multivariate Cox regression was conducted. GWI and GCW were the independent factors for predicting MACE after adjusting the significant parameters shown in Table 3. However, LV-EF and GLS were not independent variables when GWI or GCW was included in the model (Fig. 4). Adding GLS to models that included significant variables did not increase the power of the model (**χ**^**2**^ difference = 1.7, *p* > 0.05). Nevertheless, if GWI (**χ**^**2**^ difference = 13.3, *p* < 0.001) or GCW (**χ**^**2**^ difference = 7.4, *p* < 0.01) was included in the model, it increased the power of the model.

**Table 2:** Univariate Cox regression analysis to identify the determinants of MACE

Parameters	HR	95% CI	*P*
**Baseline characteristics**			
Age, years	0.998	0.974–1.023	0.88
Male, %	0.442	0.223–0.875	0.019
BMI, kg/m^2^	0.967	0.877–1.066	0.50
SBP, mm Hg	0.978	0.958–0.998	0.028
DBP, mm Hg	1.022	0.939–1.004	0.09
NYHA class, ≥II	2.728	1.234–6.029	0.013
**Clinical chemistry**			
NT-proBNP > 681, pg/mL	3.550	1.691–7.451	0.001
hs-TNT > 34, ng/L	2.457	1.192–5.066	0.015
**Echocardiography**			
LV-EDD, mm	1.157	1.016–1.105	0.007
LV-ESD, mm	1.131	1.028–1.097	< 0.001
LV-EDV, mL	1.011	1.005–1.017	0.001
LV-ESV, mL	1.028	1.008–1.021	< 0.001
LV-EF, %	0.927	0.895–0.959	< 0.001
IVS, mm	0.853	0.721–1.010	0.07
PW, mm	0.934	0.782–1.116	0.45
LV mass index, g/m^2^	1.009	1.000-1.018	0.06
MAPSE, cm	0.169	0.053–0.539	0.003
LV-GLS, -%	0.876	0.761–0.902	< 0.001
GWI, mm Hg%	0.998	0.997–0.999	< 0.001
GCW, mm Hg%	0.998	0.997–0.999	< 0.001
GWW, mm Hg%	1.002	0.999–1.005	0.19
GWE, %	0.932	0.904–0.962	< 0.001
E/e’	1.085	1.034–1.139	0.001
LAVI, mL/m^2^	1.018	1.001–1.036	0.040
RV-EDD, mm	1.008	0.960–1.059	0.74
RV-EDA, cm^2^	1.042	0.976–1.113	0.22
RV-ESA, cm^2^	1.088	1.004–1.179	0.040
FAC, %	0.202	0.014–2.836	0.24
RV-GLS, -%	0.888	0.827–0.953	0.001
TAPSE, cm	0.476	0.238–0.954	0.036
RA-ESA, cm^2^	0.993	0.931–1.059	0.83
RA-AI, cm^2^/m^2^	1.041	0.923–1.175	0.51

Table notes identical to Table 1

**Table 3:** Multivariate Cox regression models to predict MACE

Parameter	HR	95% CI	*P*
**Model 1: Baseline (χ** ^**2**^ ** = 26.4)**			
NYHA class, ≥II	1.588	0.676–3.731	0.29
NT-proBNP > 681, pg/mL	1.868	0.802–4.353	0.15
E/e’	1.028	0.967–1.093	0.38
LV-EF, %	0.952	0.912–0.993	0.022
**Model 2: Baseline + GLS (**χ ^**2**^ **= 28.1,*p*** **>0.05)**			
NYHA class, ≥II	1.508	0.633–3.595	0.35
NT-proBNP > 681, pg/mL	1.811	0.765–4.288	0.18
E/e’	1.023	0.958–1.093	0.49
LV-EF, %	0.972	0.917–1.030	0.34
GLS, -%	0.931	0.818–1.061	0.28
**Model 3: Baseline + GLS + GCW < 1,238 mm Hg% ( χ**^**2**^ **= 35.5,*p* < 0.01)**			
NYHA class, ≥II	1.705	0.725–4.007	0.22
NT-proBNP > 681, pg/mL	1.811	0.765–4.288	0.18
E/e’	1.033	0.961–1.111	0.38
LV-EF, %	0.976	0.919–1.035	0.42
GLS, -%	1.050	0.903–1.223	0.53
GCW < 1,238 mm Hg%	4.461	1.533–12.979	0.006
**Model 4: Baseline + GLS + GWI < 788 mm Hg% ( χ**^**2**^ **= 41.4,*p* < 0.001)**			
NYHA class, ≥II	1.920	0.789–4.673	0.15
NT-proBNP > 681, pg/mL	1.268	0.499–3.223	0.62
E/e’	1.014	0.935-1.100	0.73
LV-EF, %	0.967	0.909–1.030	0.30
GLS, -%	1.071	0.912–1.258	0.40
GWI < 788 mm Hg%	5.460	1.663–17.923	0.005

MACE, major adverse cardiac events; NYHA, New York Heart Association; NT-proBNP, N-terminal pro brain natriuretic peptide; hs-TNT, high-sensitive cardiac troponin T; E/e’, ratio of mitral inflow velocity (E) to tissue Doppler mitral annular velocity (e’); LAVI, left atrial volume index; LV, left ventricular; EF, ejection fraction, biplane Simpson’rule; GLS, global longitudinal strain; GWI, global work index; GCW, global constructive work

Integrated discrimination improvement and the Harrell C concordance statistics are shown in Table 4. Both GLS and LV-EF had similar concordance statistics. However, GWI or GCW had higher concordance statistics than LV-EF and GLS. The model with baseline and GLS plus GWI or GCW had higher concordance statistics than the baseline model and GLS. Compared with the model of Baseline and GLS, the IDI of baseline and GLS plus GWI was 0.254, which means the accuracy of predicting MACE increased by 25.4% (*p* = 0.02). Moreover, the IDI of baseline and GLS plus GCW was 0.239, which means the accuracy of predicting MACE increased by 23.9% (*p* = 0.04).

**Table 4:** IDI and C-index to proof incremental values of myocardial work indices

Parameter	C-index	95% CI	IDI
NYHA class, ≥II	0.616	0.540–0.692	
NT-proBNP > 681, pg/mL	0.617	0.526–0.707	
hs-TNT > 34, ng/L	0.586	0.508–0.664	
E/e’	0.677	0.585–0.769	
LAVI, mL/m^2^	0.643	0.556–0.730	
LV-EF, %	0.694	0.603–0.784	
GLS, -%	0.692	0.589–0.795	
GWI < 788 mm Hg%	0.700	0.618–0.782	
GCW < 1,238 mm Hg%	0.700	0.617–0.782	
Baseline^a^	0.726	0.637–0.814	
Baseline^a^ + GLS^b^	0.726	0.633–0.819	0.154
Baseline^a^ + GLS^b^ + GWI^c^	0.769	0.682–0.856	0.254
Baseline^a^ + GLS^b^ + GCW^d^	0.755	0.668–0.841	0.239

a: Baseline model includes NYHA class (≥ II), NT-proBNP > 681, hs-TNT > 34, E/e’, LAVI, LV-EF. NYHA, New York Heart Association; NT-proBNP, N-terminal pro brain natriuretic peptide; hs-TNT, high-sensitive cardiac troponin T; E/e’, ratio of mitral inflow velocity (E) to tissue Doppler mitral annular velocity (e’); LAVI, left atrial volume index; LV, left ventricular; EF, ejection fraction, biplane Simpson’s rule; GLS, global longitudinal strain; GWI, global work index; GCW, global constructive work. IDI, integrated discrimination improvement; C-index, the Harrell C concordance statistics. b: *P* = 0.269 vs. Baseline model. c:*P* = 0.02 vs. Baseline + GLS model. d: *P* = 0.04 vs. Baseline + GLS model

Intra- and interobserver variabilities of myocardial work indices are shown in the supplementary table.

## Discussion

Our study analyzes the association between myocardial work indices and MACE in DCM patients. We found that (1) GWI and GCW were not only independent factors for predicting MACE, but also the strongest parameters associated with worse long-term outcomes; (2) myocardial work indices provide incremental values to LV-EF and LV-GLS for predicting MACE.

Previous study showed that GWI (HR for every 50 mm Hg% = 0.85, 95%CI 0.77–0.94, *p* = 0.002) as well as GCW (HR for every 50 mm Hg% = 0.86, 95%CI 0.79–0.94, *p* = 0.001) were independent predictors of MACE in patients with advanced heart failure [17]. Similarly, Wang et al. demonstrated that MW indices provided incremental prognostic values over GLS and LV-EF regarding all-cause mortality (HR 1.23, 95%CI 1.04–1.46, *p* = 0.015) or HF hospitalization (HR 1.12, 95%CI 1.03–1.25, *p* = 0.012) in patients with heart failure and reduced ejection fraction (HFrEF), this being consistent with our findings [18].

In addition, besides LV-EF and LV-GLS, GCW and GWI showed the strongest association with MACE. Patients with GCW < 1,238 mm Hg% showed significantly worse outcomes compared to those with higher GCW. In a previous study, GCW has been validated to have a higher accuracy, sensitivity and specificity than GLS for predicting myocardial fibrosis in patients with DCM [19]. Centurión et al. demonstrated that myocardial fibrosis was associated with higher risk for mortality, arrhythmia events, and sudden death, which may explain the results of our study and provide additional value for clinicians to request the assessment of myocardial work indices by strain imaging echocardiography [20]. Similarly, another study validated that GCW was the only predictor of MACE after adjusted to LV size, LV-EF and GWE in patients with HFrEF. Moreover, GCW < 910 mm Hg% had a higher risk of MACE (HR 11.09, 95%CI 1.45–98.94, *p* = 0.002) [21].

In our study, the AUC of GWI was 0.76, representing the main predictor of worse long-term outcomes along with GCW, with corresponding cut-off value of 788 mm Hg%, sensitivity of 64.7% and specificity of 82.9%. GWI < 788 mm Hg% had a higher risk of MACE than patients with GWI ≥ 788 mm Hg%. Hedwig et al. demonstrated that GWI had a significant correlation with peak oxygen consumption (peak VO_2_) and NT-proBNP [22]. While previous studies showed that peak VO_2_ and NT-proBNP were relevant prognostic factors in patients with heart failure [23, 24]. DCM is a progressive disease that eventually evolves to end-stage heart failure or death. These findings may imply that MW indices have a potential prognostic value in patients with DCM. Moreover, Cui et al. demonstrated that comparing to condition before therapy, GWI and 6-minute walk distance were significantly increased whereas GLS and LV-EF showed no significant changes in DCM patients after 6 months therapy, suggesting that MW indices were more sensitive in detecting the improvement of cardiac function and may provide an additional value for assessing therapy effect [25].

### Clinical relevance

MWIs allow the comprehensive assessment of LV performance and may have several potential advantages over traditional measures of LV function, such as EF, which may be normal at early stages of heart disease. MWIs may be more sensitive than EF for detecting early changes in LV function, they may also be less load-dependent. In DCM, GWI and GCW are indices which seem to provide the most valuable information. Furthermore, MWIs show promise in predicting cardiotoxicity early during chemotherapy, and has been validated in various cardiac pathologies, including coronary artery disease, heart failure, and CRT-response prediction [8].

### Limitations

There are several limitations in the present study. Firstly, this was a single-center, small-sample, retrospective study. Larger prospective studies are needed to validate the clinical utility and prognostic implications of the MW indices. Secondly, due to the limited sample size and number of events, we were hindered to adjust for all potential confounders in the multivariable Cox regression. Finally, some patients were excluded due to poor deformation imaging quality, which may affect the actual MACE rate of patients with DCM in our collective.

## Conclusions

In our study, the novel MW indices derived from non-invasive left ventricular pressure-strain loops integrating GLS and blood pressure showed prognostic relevance in DCM patients. GWI and GCW may have an additional value beyond LV-EF and LV-GLS for predicting adverse outcomes in DCM. Further studies are needed to validate our findings in different settings.

### Electronic supplementary material

Below is the link to the electronic supplementary material.

Supplementary Material 1

## Data Availability

The data that support the findings of this study are available on request from the corresponding author.

## Abbreviations

DCM: dilated cardiomyopathy
EF: ejection fraction
GCW: global constructive work
GLS: global longitudinal strain
GWE: global work efficiency
GWI: global work index
GWW: global wasted work
hs-TNT: high-sensitive cardiac troponin T
IDI: integrated discrimination improvement
LV-PSL: left ventricular pressure-strain loop
MACE: major adverse cardiac events
MWI: myocardial work indices
NT-proBNP: N-terminal pro brain natriuretic peptide
NYHA: New York Heart Association
RV-GLS: right ventricular global longitudinal strain
