# Supplementary material for: Prognostic relevance of global work index and global constructive work in patients with non-ischemic dilated cardiomyopathy
Source: Int J Cardiovasc Imaging. 2024 May 23;40(7):1575–84. doi: 10.1007/s10554-024-03144-5 (PMC11258082; doi:10.1007/s10554-024-03144-5)
Supplement: Supplementary file 1 — Supplementary Material 1 [file 10554_2024_3144_MOESM1_ESM.docx]

**Supplementary table.** Inter- and intra-observer variability of myocardial work indices

| **Parameters** | **Inter-observer** | | **Intra-observer** | |
| --- | --- | --- | --- | --- |
|  | **ICC** | **95% CI** | **ICC** | **95% CI** |
| GWI, mm Hg% | 0.979 | 0.948-0.992 | 0.987 | 0.966-0.995 |
| GCW, mm Hg% | 0.980 | 0.945-0.992 | 0.977 | 0.931-0.992 |
| GWW, mm Hg% | 0.842 | 0.604-0.937 | 0.951 | 0.878-0.980 |
| GWE, % | 0.967 | 0.917-0.987 | 0.948 | 0.861-0.980 |

GWI, global work index; GCW, global constructive work; GWW, global wasted work; GWE, global work efficiency; ICC, intraclass correlation coefficients.
